# Self-Regulation and Shame as Mediators Between Childhood Experiences and Young Adult Health

**DOI:** 10.3389/fpsyt.2021.649911

**Published:** 2021-04-16

**Authors:** Elizabeth Mathews Rollins, AliceAnn Crandall

**Affiliations:** Department of Public Health, Brigham Young University, Provo, UT, United States

**Keywords:** adverse childhood experiences, positive childhood experiences, self-regulation, shame, structural equation model, young adult, depression, anxiety

## Abstract

The purpose of this study was to determine whether self-regulation and shame mediated the relationship between adverse and positive childhood experiences (ACEs and PCEs) and young adult health. Data came from the Flourishing Families Project (FFP), a 10-year longitudinal study. Adolescent participants (*N* = 489; 51% female) completed an annual survey. Data were analyzed using structural equation modeling. Results indicated that PCEs negatively predicted shame and positively predicted self-regulation while ACEs negatively predicted self-regulation. Shame mediated the relationship between PCEs and depression. Self-regulation mediated the relationship between both ACEs and PCEs with anxiety; self-regulation also mediated the relationship between ACEs and substance abuse. Childhood experiences appear to affect the development and maintenance of self-regulation in adolescence. Self-regulation appears to be especially important in protecting against depression, anxiety, and substance abuse in young adulthood.

## Introduction

Adverse childhood experiences, or ACEs, are potentially traumatic and stressful events that occur before the age of 18 such as emotional, physical, or sexual abuse, and household dysfunction (e.g., parental depression, incarceration of a family member, family member using illicit drugs) (1). ACEs are associated with negative health behaviors and outcomes such as alcohol use, smoking, and depression (1–3). Conversely, cumulative scores of advantageous factors known as positive childhood experiences (PCEs; also referred to as advantageous childhood experiences and benevolent childhood experiences in the literature) are associated with lower depression and stress and better positive psychology, irrespective of ACE scores (4). PCEs include healthy relationships with others such as feeling safe with at least one parent, having good neighbors, feeling like one's teachers care about them, and having at least one good friend. In the absence of these positive relationships, higher levels of distress have been reported among youth (5), but when present, PCEs may attenuate the negative effects of ACEs on health (4, 6–9).

In the current study, we examined the role of two potential mediators, self-regulation and shame. These mediators were selected because their healthy development may be influenced by childhood experiences that occur during adolescence and they are known to be associated with adult psychopathology (10, 11). Self-regulation is the ability to engage in goal-directed behavior through controlling one's behaviors, emotions, and thoughts (12). Some research has shown that shame is an aspect of emotional self-regulation; shame has been defined as a family of emotions such as embarrassment, inferiority, and humiliation that one has internalized that are the result of others' negative evaluations (13, 14).

### Childhood Experiences and Young Adult Mental Health

A growing number of studies indicate that ACEs experienced during the first 18 years of life increase mental health problems in young adulthood. For example, one study comprised from a British cohort born during a 1-week period in 1958 in England, Scotland, and Wales found that ACEs (measured at ages 7, 11, and 16) predicted worse young adult mental health (15). Among 239 college students, with a mean age of 20 years old, from a large public university in the Midwest, ACEs that occurred before age 18 years predicted increased likelihood of developing depression or an anxiety disorder over the duration of the semester (16). Another study of 1,578 high school seniors from nine Boston Metropolitan area public schools found that cumulative adversity resulted in disproportionately worse mental health in young adulthood based on participant lifetime reporting (17). Similarly, a study comprised 1,142 participants of the Chicago Longitudinal Study (CLS) aged 22–24 found that ACEs scores were related to poorer self-rated health and more depressive symptoms and substance abuse in early adulthood (18).

Fewer studies have examined the relationship between PCEs and young adult health. Of the studies that have investigated this relationship, the majority have focused on individual positive experiences rather than cumulative PCE scores. For example, prior research has shown that positive relationships such as the parent-child relationship lead to the development of better cognitive and emotional development (19, 20). However, taken together these studies indicate that PCEs protect against psychological stress in early and later adulthood (7, 21).

Some research suggests that experiences in adolescence could be more predictive of young adult health than positive and adverse experiences that occurred in early or middle childhood (22). However, most research examining the effects of ACEs on young adult health has examined childhood experiences that occurred between birth and age 18 years. Research examining ACEs and PCEs at specific points during childhood and adolescence is important to better understand the relative impact of these experiences at each stage on child and youth development. Furthermore, there is a need for studies that explain the mechanisms through which childhood experiences affect lifelong health.

### The Effects of Adverse and Positive Experiences on Adolescent Development

Adolescence and young adulthood are important to study because adolescence is an important period for social and cognitive development (23), and this development continues into young adulthood when individuals undergo key life transitions such as leaving home, entering the workforce, beginning college, or getting married. Given all these major life transitions, mental health issues often begin in late adolescence and young adulthood (24). Adolescence is a key sensitive period for the development of the prefrontal cortex, which develops over the first three decades of life (25, 26). The brain's prefrontal cortex governs, in part, self-regulation, and shame (27). Due to the protracted development of the prefrontal cortex, proximal environmental factors such as family experiences during adolescence play a key role in the healthy development of self-regulation and shame (28). ACEs and PCEs in adolescence may affect the structure and function of the brain as well as neural communication between different parts of the brain associated with self-regulation and shame (25, 29, 30). Brain imaging studies indicate that ACEs may negatively affect the development and structure of the prefrontal cortex, hippocampus, amygdala, and other parts of the brain that are important to self-regulation and emotion, whereas PCEs, such as positive parenting, promote more normative brain development even in the face of ACEs (20, 31).

### Self-Regulation and Shame as Potential Mediators Between Childhood Experiences and Young Adult Health

In the context of this study, if an individual is exposed to forms of abuse or maltreatment in adolescence, the development of their self-regulatory skills and shame management could be poorly impacted. Higher shame and impaired self-regulatory skills may negatively affect young adult capacity and confidence to manage life's challenges and early symptoms of psychopathology, and possibly lead to unhealthy coping mechanisms such as substance abuse. On the other hand, if an individual experiences a variety of positive experiences, this could enhance the development of self-regulation and reduce internalizing of unhealthy emotions associated with shame. Well-developed self-regulatory capacities provide tools to aid in key life changes occurring in young adulthood, leading to more fulfilling experiences in young adulthood that may help to combat substance abuse and symptoms associated with anxiety and depression (32, 33). In fact, individuals who experience a high number of PCEs may be able to overcome the negative effects of adversity, yielding better mental health as young adults regardless of ACEs score (31, 34).

Existing research lends support for this framework and suggests that self-regulation and shame may mediate the relationship between ACEs and PCEs with young adult mental health. Higher ACE scores have been linked to worse self-regulation during adolescence (35), while higher PCEs are associated with better self-regulation (4). Better self-regulation leads to less depressive symptoms, even in the presence of ACEs (36, 37). As it relates to shame, exposure to childhood trauma has been associated with increased shame in multiple adolescent populations (38–40), leading to higher rates of depression in young adulthood (41, 42).

### Aims and Hypotheses

Extant literature indicates that childhood experiences affect self-regulation and shame (42), and that self-regulation (43) and shame (44) affect later health. However, most prior studies have been cross-sectional and to our knowledge research has not yet examined shame and self-regulation as mediators between childhood experiences with young adult health. Understanding the mechanisms through which childhood experiences that occur during adolescence affect later health may be important to developing more effective interventions. Furthermore, few studies have examined both ACEs and PCEs in the same model as predictors of young adult health. The purpose of this study was to address this gap in the literature by examining the potential mediating pathways of shame and self-regulation between both PCEs and ACEs on young adult mental health. We hypothesized that both shame and self-regulation would mediate the relationship between ACEs and PCEs with depression, anxiety, and substance abuse. Specifically, we predicted that (1) ACEs would decrease self-regulation and increase shame, (2) PCEs would increase self-regulation and decrease shame, (3) higher self-regulation would protect against poor mental health, and (4) greater shame would increase poor mental health.

## Methods

### Sample and Procedures

The data for this study came from the Flourishing Families Project (FFP), a 10-year longitudinal study with annual follow-ups that took place in a large city in the Northwestern United States. The FFP was approved by the university Institutional Review Board (IRB). Baseline data were collected among 500 adolescents and their parents in 2007, when adolescents were between the ages of 10 and 13 years. Participants were initially recruited from the Polk Directory National Database, a registry based on telephone, magazine, and internet subscriptions. As participants did not proportionally represent those of lower socioeconomic statuses, an additional low-income sample was recruited *via* referrals and fliers, resulting in a sample more representative of local zip codes.

In each wave (spaced 1 year apart), adolescents and their parents completed a survey. Data for waves 1–5 were collected through home surveying; as participants transitioned into adulthood, in waves 6–10 the survey was administered online. Retention was high across waves, with 87.6% of the sample participating in wave 10. As a result of some missing demographic information, data from 489 of the original 500 adolescents and their parents were used in the current study.

### Measures

#### Independent Variables: Childhood Experiences

An ACE questionnaire was not included in the FFP. However, items that fit the criteria of an ACE were adapted to create an ACEs measure, which has been further described elsewhere (7). A total of eight ACEs were included: poverty (parent report), mental illness in one or both parents (parent report), substance abuse in the household (adolescent report), incarcerated household member (adolescent report), parental separation or divorce (parent report), interparental conflict (adolescent report), child physical punishment (adolescent report), and child emotional abuse (adolescent report). Items were reported in waves 1–5 when adolescents were ~13–18 years. Some ACEs were based on a single item (e.g., parent trouble with the law—reports of sometimes or higher were coded as an ACE) while other ACEs were based on a scale or questionnaire (e.g., Center for Epidemiologic Studies Depression Scale to measure parental depression; average score of sometimes or higher were coded as an ACE). If the ACE occurred in any wave, it was coded as a one for being present. If the ACE did not occur in any wave, it was coded as zero. The scores were summed for a cumulative ACE score ranging from 0 to 8.

A PCE questionnaire was also not included in the FFP. However, items that fit the criteria of a PCE were used to create a PCE score (7). A total of 10 PCEs were included including adolescent report of parent child connection, parent report of parental monitoring, having at least one good friend (adolescent report), having beliefs that gave you comfort (adolescent report), liking school (adolescent report), having at least one teacher who cares about you (adolescent report), having good neighbors (parent report), having opportunities to have a good time (adolescent report), liking or feeling comfortable with yourself (adolescent report), and having a predictable home routine that included things like a regular bedtime and meals (parent report). Some PCEs were based on single items in the survey (e.g., agreement or strong agreement with the statement “I feel happy in school”) and others were based on a survey scale (e.g., Rosenberg Self-Esteem scale, with average scores indicating agreement or higher marked as a PCE). If a PCE occurred in any wave, it was coded as one for being present. If the PCE did not occur in any wave, it was coded as zero. The scores were summed for a total PCE score ranging from 0 to 10.

#### Mediating Variables: Self-Regulation and Shame

Self-regulation was measured in wave 7 from a self-regulation scale developed by Novak and Clayton (45). The 13-item scale included response options ranging from 1 = never true to 4 = always true. The scale is composed of behavioral, emotional, and cognitive self-regulation subscales, but only subscales for behavioral and emotional self-regulation were used in the current study. Sample items included: “I get distracted by little things” and “I have a hard time controlling my temper.” Self-regulation was calculated based on both adolescent self-report and parent report of adolescent self-regulation. To facilitate parent report about their child, the items were adapted (e.g., “My child gets distracted by little things.”). Each subscale was summed and averaged for each reporter, resulting in six subscale scores. The Cronbach's alphas for each subscale and reporter ranged from 0.75 to 0.90. Prior studies using the self-regulation scale among children only found Cronbach's alphas >0.90 (45). Prior studies have examined the factor structure of self-regulation combining parent and child report and have found good fit in confirmatory factor analysis (46).

Shame was measured in wave 7 *via* adolescent self-report using the 8-item Shame Profile Scale (47). Response options were on a 5-point Likert Scale ranging from 1 = never to 5 = almost always, with higher scores indicating greater shame. Sample items included: “I feel like I am never quite good enough” and “I think that people look down on me.” The Cronbach's alpha for the current study was 0.96, which is higher than reliabilities from prior studies ranging from 0.75 to 0.80 (47).

#### Dependent Variables: Mental Health Indicators

The self-reported mental health indicators of depression, anxiety, and substance abuse were measured in wave 10 when participants were between 20 and 23 years. Depression was measured using the 20-item Center for Epidemiological Studies Depression Scale for Children (48), and had a Cronbach's alpha of 0.92 in the current sample. Response options ranged from 1 = not at all to 4 = a lot. Higher scores indicated more depressive symptoms. Sample items included: “I felt down and unhappy” and “I felt like I was too tired to do things.”

Higher scores on the Spence Child Anxiety Inventory (49), a 6-item scale with a Cronbach's alpha of 0.89 in the current study, indicated greater anxiety among participants. Response options were on a 4-point scale, ranging from 0 = never to 3 = always. Sample items included: “When I have a problem, my heart beats really fast” and “I worry that something bad will happen to me.”

The Adolescent Alcohol and Drug Involvement Scale (50) was used to measure substance abuse. The 6-item scale had a Cronbach's alpha of 0.80 in the current sample. Because most of the participants could legally drink alcohol in wave 10, alcohol use was excluded. The frequency of use of marijuana, tobacco, other illegal drugs, legal drugs without a prescription, and binge drinking were measured with an 8-point Likert scale, ranging from never to several times a day. Due to low counts on some responses, categorization of binge drinking was 0 = never, 1 = several times a year, and 2 = monthly or more. Categorization of other substances was 0 = never used, 1 = tried but quit, and 2 = currently use.

#### Control Variables

Because there were socioeconomic differences in sampling methodology, we controlled for sampling (1 = Polk sample; 0 = convenience sampling) in our models. Adolescent gender, age in years at baseline, and race were also included as control variables in the final models. Gender was measured as 1 = male and 2 = female among adolescents. Race was coded as 0 = other race; 1 = Black/African American race; 2 = multi-racial; 3 = Asian; 4 = non-Hispanic White. Race was included as a covariate because prior studies have indicated that non-Hispanic Whites and Asians have lower risk for ACEs as compared to African Americans and other minority races (51, 52).

### Statistical Analysis

Data were analyzed in Mplus version 7. A measurement model was established by conducting confirmatory factor analysis (CFA) to create latent variables for the three outcome constructs (depression, anxiety, and substance abuse) and the two mediators (self-regulation and shame). Items with factor loadings <0.40 were dropped. Both parent and adolescent-report of cognitive self-regulation were dropped due to low loadings as was parent report of adolescent behavioral self-regulation. Model fit for the final measurement model was adequate (RMSEA = 0.05; CFI = 0.95).

A structural equation model (SEM) was fit by regressing the three mental health outcomes on shame, self-regulation, ACEs, and PCEs. Shame and self-regulation were also regressed on ACEs and PCEs. Next, the control variables were added by regressing the outcome, mediating, and independent variables on the controls. To ensure robust standard errors, we used 5,000 bootstraps to test the significance of indirect effects (53).

All models were estimated using variance-adjusted weighted least squares approximation, which is appropriate for categorical data. Missing data were minimal and were accounted for in Mplus using Full Information Maximum Likelihood (FIML). For both the measurement and structural model, model fit was determined based on the Root Mean Square Error of Approximation (RMSEA; values <0.08 indicated adequate fit) and Comparative Fit Index (CFI; values >0.90 indicated adequate fit) (54).

## Results

### Demographics

On average, adolescents were 11.3 years old at age baseline, with 51% of participants who reported their gender as female and 49% reported male. Reported race distribution was as follows: 69.7% non-Hispanic White, 12.9% as African American or Black, 11.2% as multi-racial, 3.9% as Asian, and 2.3% as other race. The average ACE score for adolescents was 2.7, and the average PCE score was 8.2. Table 1 includes a correlation matrix of all model variables.

**Table 1:** Correlation matrix, *N* = 489.

	**ACE**	**PCE**	**Self-regulation**	**Shame**	**Depression**	**Anxiety**	**Substance abuse**	**Age at baseline**	**Race**	**Gender**	**Sampling method**
ACE	1.000										
PCE	−0.255^***^	1.000									
Self-regulation	−0.265^***^	0.256^***^	1.000								
Shame	0.054	−0.190^***^	−0.648^***^	1.000							
Depression	0.116^*^	−0.120^*^	−0.457^***^	0.474^***^	1.000						
Anxiety	0.110^*^	−0.100^*^	−0.511^***^	0.379^***^	0.575^***^	1.000					
Substance abuse	0.072	−0.120^*^	−0.173^**^	0.071	0.104	0.102	1.000				
Age at baseline	0.171^***^	−0.155^***^	0.143^**^	−0.041	−0.080	−0.062	0.043	1.000			
Race	−0.389^***^	0.198^***^	0.130^*^	0.040	−0.018	−0.035	0.093	−0.089	1.000		
Gender	0.015	−0.006	−0.242^***^	−0.207^***^	0.196^***^	0.315^***^	−0.198^***^	−0.033	−0.030	1.000	
Sampling method	0.225^***^	−0.023	0.084	−0.094^*^	−0.017	−0.072	−0.084	0.080	−0.372^***^	−0.057	1.000

* *p < 0.05*.

** *p < 0.01*.

*** *p < 0.001*.

*For gender, 0 = male, 1 = female*.

### Self-Regulation and Shame as Mediators Between Childhood Experiences and Young Adult Mental Health

**Figure 1:**
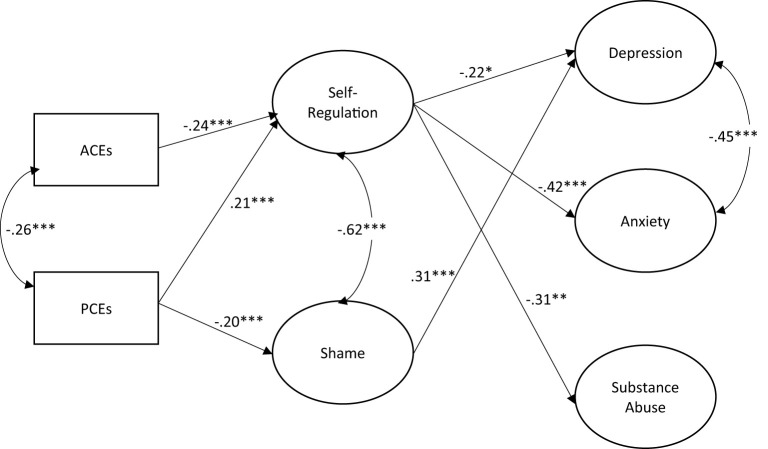
Structural equation model for the role of childhood experiences oh shame, self-regulation, and young adult mental health, *N* = 489. *p < 0.05. **p < 0.01. ***p < 0.001. Model fit: RMSEA = 0.049; CFI = 0.946. Model controls for adolescent race, gender, age at baseline, and sampling method. Path coefficients are standardized. Only significant paths are shown.

Table 2 contains the structural equation modeling results for the role of childhood experiences on shame, self-regulation, and young adult mental health (Figure 1 provides the standardized results of the final model with significant paths only shown). Neither ACEs nor PCEs directly predicted any of the young adult mental health outcomes. PCEs negatively predicted shame and positively predicted self-regulation. ACEs did not predict shame but were negatively associated with self-regulation. Shame predicted more depression but was not associated with anxiety or substance abuse. Self-regulation predicted less depression, anxiety, and substance abuse.

**Table 2:** Structural equation model results for the role of childhood experiences on shame, self-regulation, and young adult mental health (*N* = 489; model fit: RMSEA = 0.049; CFI = 0.946).

	**Shame**	**Self-regulation**	**Depression**	**Anxiety**	**Substance abuse**
ACEs	0.038	−0.235^***^	0.042	−0.001	0.028
PCEs	−0.204^***^	0.208^***^	0.003	0.003	−0.075
Shame	–	–	0.311^***^	0.066	−0.098
Self-Regulation	–	–	−0.223^*^	−0.419^***^	−0.312^**^

* *p < 0.05*.

** *p < 0.01*.

*** *p < 0.001*.

*Model controls for adolescent race, gender, age at baseline, and sampling method. Coefficients are standardized*.

Table 3 shows the significant mediating pathways based on 5,000 bootstrap computations. Shame partially mediated the relationship between PCEs and depression. Self-regulation partially mediated the relationship between both ACEs and PCEs with anxiety. Self-regulation also partially mediated the relationship between ACEs and substance abuse. No other mediating pathways were significant.

**Table 3:** Results of significant paths for shame and self-regulation as mediators.

**Significant indirect pathways**	**Z-score**
PCEs > shame > depression	−3.011^**^
PCEs > self-regulation > anxiety	−2.530^*^
ACEs > self-regulation > anxiety	2.788^**^
ACEs > self-regulation > substance abuse	2.202^*^

* *p < 0.05*.

** *p < 0.01*.

## Discussion

The purpose of this study was to determine whether self-regulation and shame mediated the relationship between ACEs and PCEs with young adult mental health. Prior research has indicated that higher ACE scores lead to worse self-rated health, more prevalent depressive symptoms, and substance abuse (18). However, less is understood about the mechanisms through which childhood experiences affect young adult health. We hypothesized that childhood experiences would work through shame and self-regulation to affect young adult mental health. The results of this study largely confirmed our hypothesis. Both ACEs and PCEs predicted self-regulation in the expected direction, with self-regulation mediating the relationships between both ACEs and PCEs with anxiety and between ACEs and substance abuse. PCEs but not ACEs predicted shame, and shame mediated the relationship between PCEs and depression.

The results of the study are consistent with extant literature relating to how childhood experiences may affect the development of shame, self-regulation, and later mental health. Shame and self-regulation are developing at the same time as the occurrence of many key positive and adverse adolescent experiences. One explanation for why ACEs were predictive of worse self-regulation and PCEs were associated with less shame and better self-regulation is that cumulative adversity or advantage during adolescence may affect the connections made by neurons in the brain (30), thereby affecting self-regulatory skills and expressions of shame. Our findings are consistent with cognitive science (22, 55) that indicates that ACEs impact the brain's structure including the growth of the prefrontal cortex, hippocampus, amygdala, and the superior parietal region of the brain (20, 31). Conversely, individual PCEs such as positive parenting have been shown to promote healthier development of the amygdala and prefrontal cortex, especially in the face of adversity (20, 31). Because PCEs promote healthy brain development, individuals with high PCEs appear to have stronger self-regulation and less shame, leading them to more effectively control their thoughts, behaviors, and emotions, leading to better health in adulthood including lower rates of mental illness and less substance use.

Beyond cognitive science, discussion regarding how self-regulation and shame may affect later mental health is merited. Given the key life transitions happening in young adulthood and the increase in stressors as young adults juggle new life demands, shame and self-regulatory skills may be particularly important to managing mental health. In the current study, shame was predictive of more depression only while self-regulation was associated with less depression, anxiety, and substance abuse. Shame is inclusive of a range of negative self-appraisals that give way to more acute negative feelings such as self-contempt that have been widely associated with increased depression and anxiety symptoms (10). Surprisingly, we did not find an association between shame and anxiety. This may have been due, in part, to the greater relative importance of self-regulation. As self-regulation involves controlling emotions, behaviors, and cognitions, deficits in self-control mean lower levels of self-control and higher levels of impulsivity that have been linked with riskier behaviors such as substance use, particularly among individuals with a history of experiencing maltreatment (11). Self-regulatory capacities are not just important to externalizing behaviors such as substance use, but they are also important in helping individuals to regulate their emotions and cognitions, including cognitive distortions that are associated with psychopathology (56). Thus, considerations of self-regulation may be particularly important when examining how childhood experiences affect later mental health.

It is noteworthy that in pairwise correlations in the current study, PCEs were more strongly associated with both self-regulation and shame than were ACEs. Additionally, in the final models, PCEs were associated with depression through shame while ACEs had no direct nor indirect effect on depression. However, ACEs, but not PCEs, were indirectly associated with substance use. Thus, PCEs may have a greater impact on internalizing symptoms such as depression in young adulthood as compared to ACEs. However, externalizing symptoms such as substance abuse may be a way for individuals with ACEs to cope with the trauma that they have experienced.

### Implications for Practice

These findings have important implications for practice. Preventing ACEs should always be a goal in public health. However, given the high prevalence of some ACEs (e.g., mental health in parents and parental separation and divorce) it is not feasible to always prevent ACEs. Therefore, consideration should also be given to increasing children's PCEs, which may be important to the development and maintenance of cognitive capacities such as the regulation of emotion and behaviors, which improve lifelong health (57). Programs designed to promote mindfulness and self-compassion may help to reduce shame (58). Several interventions have been successful at increasing self-regulation through enhancing positive experiences during middle childhood and adolescence (59). For example, the Family Check-Up has been found to increase youth's self-regulation, leading to less substance abuse, depression, and behavioral problems and better school engagement among youth (60, 61). Similarly, the Student Success Skills Program has been found to improve self-regulation in school settings (62).

### Strengths and Limitations

A few limitations in the current student are important to note. We used proxy measures of ACEs and PCEs that have been used in prior research [e.g., see (7)] and closely match the questions in traditional ACEs and PCEs scales. Even though the concepts matched, caution is warranted when comparing results to studies that used actual ACE and PCE scales. We also used shame as a general measure and did not use a specify type such as internal or external shame. Our lack of a relationship between ACEs and shame may have been due to the lack of specificity in our shame measure. Furthermore, a more focused shame measure may help to lend better specificity of the shame mechanism through which childhood experiences affect later health. Thus, further research examining different aspects of shame is warranted. The Flourishing Families Project was focused on adolescent participants and we were unable to assess ACEs and PCEs that occurred before adolescence. Our results do highlight the effects of childhood experiences that occur during adolescence on self-regulation and shame, which is an important development period to understand. ACEs and PCEs experienced in early or middle childhood may affect brain development in different ways. Thus, the mechanisms through which childhood experiences that occur in early/middle childhood affect young adult health may be different from what was found in our study. Additionally, we did not have data on shame, self-regulation, or the mental health outcomes before adolescence, and the temporality of associations in our final model are not definitive.

Despite these limitations, this study has several strengths. Generally, surveys that measure ACEs and PCEs are administered in adulthood, years after the events have occurred, thereby resulting in recall bias of childhood experiences. In the current study, adolescents reported on their experiences each year, which decreased the likelihood of recall bias. Additionally, some measures included multiple reporters (e.g., parents and adolescents self-report), which increased the robustness of those measures. This study addresses a gap in the literature by examining the mechanisms through which both adverse and advantageous experiences affect health during a sensitive period of social and cognitive development (adolescence) using data from a 10-year longitudinal study.

## Conclusion

Prior research has indicated a link between ACEs and worse adult health (1, 2) as well as between PCEs and better adult health outcomes (4, 6). Less is known about the mechanisms through which childhood experiences that occur specifically in adolescence affect young adult mental health. The current study indicates that ACEs may predict lower self-regulation, leading to more depression and anxiety symptoms and more substance abuse. Conversely, even in the presence of ACEs, PCEs experienced during adolescence are predictive of better self-regulation and less shame, leading to less depression and anxiety in young adulthood. Self-regulation and shame are both mechanisms through which childhood experiences may affect young adult depression, anxiety, and substance abuse, though self-regulation in particular appears to be especially salient to young adult mental health.

## Data Availability Statement

The data analyzed in this study is subject to the following licenses/restrictions: Data may be available upon request from the corresponding author and a proposal to the Flourishing Families Project principal investigators. Requests to access these datasets should be directed to ali_crandall@byu.edu.

## Ethics Statement

The Flourishing Families Project was approved by the Brigham Young University Institutional Review Board. Participants provided informed consent to participate.

## Author Contributions

ER and AC conceptualized the study. AC analyzed the data, wrote the statistical methods, and edited the entire manuscript. ER wrote the manuscript. All authors contributed to the article and approved the submitted version.

## Conflict of Interest

The authors declare that the research was conducted in the absence of any commercial or financial relationships that could be construed as a potential conflict of interest.

## References

[1] Felitti VJ Anda RF Nordenberg D Williamson DF Spitz AM Edwards V. Relationship of childhood abuse and household dysfunction to many of the leading causes of death in adults: the adverse childhood experiences (ACE) study. Am J Prevent Med. (1998) 14:245–58. 10.1016/S0749-3797(98)00017-8 9635069

[2] Merrick MT Ford DC Ports KA Guinn AS Chen J Klevens J. Vital signs: estimated proportion of adult health problems attributable to adverse childhood experiences and implications for prevention-−25 States, 2015-2017. MMWR Morb Mortal Weekly Rep. (2019) 68:999–1005. 10.15585/mmwr.mm6844e1 31697656 PMC6837472

[3] Ramiro LS Madrid BJ Brown DW. Adverse childhood experiences (ACE) and health-risk behaviors among adults in a developing country setting. Child Abuse Neglect. (2010) 34:842–55. 10.1016/j.chiabu.2010.02.012 20888640

[4] Crandall A Miller JR Cheung A Novilla LK Glade R Novilla MLB. ACEs and counter-ACEs: how positive and negative childhood experiences influence adult health. Child Abuse Neglect. (2019) 96:104089. 10.1016/j.chiabu.2019.104089 31362100

[5] Turner HA Merrick MT Finkelhor D Hamby S Shattuck A Henly M. The Prevalence of Safe, Stable, Nurturing Relationships Among Children and Adolescents. US Department of Justice, Office of Justice Programs, Office of Juvenile Justice and Delinquency Prevention (2017). Available online at: https://ojjdp.ojp.gov/sites/g/files/xyckuh176/files/pubs/249197.pdf (accessed April 6, 2021).

[6] Bethell C Jones J Gombojav N Linkenbach J Sege R. Positive childhood experiences and adult mental and relational health in a statewide sample: associations across adverse childhood experiences levels. JAMA Pediatr. (2019) 173:e193007. 10.1001/jamapediatrics.2019.3007 31498386 PMC6735495

[7] Crandall A Broadbent E Stanfill M Magnusson BM Novilla MLB Hanson CL. The influence of adverse and advantageous childhood experiences during adolescence on young adult health. Child Abuse Neglect. (2020) 108:104644. 10.1016/j.chiabu.2020.104644 32795716

[8] Narayan AJ Rivera LM Bernstein RE Harris WW Lieberman AF. Positive childhood experiences predict less psychopathology and stress in pregnant women with childhood adversity: a pilot study of the benevolent childhood experiences (BCEs) scale. Child Abuse Neglect. (2018) 78:19–30. 10.1016/j.chiabu.2017.09.022 28992958

[9] Davis JP Ports KA Basile KC Espelage DL David-Ferdon CF. Understanding the buffering effects of protective factors on the relationship between adverse childhood experiences and teen dating violence perpetration. J Youth Adolesc. (2019) 48:2343–59. 10.1007/s10964-019-01028-9 31041619 PMC6821577

[10] Callow TJ Moffitt RL Neumann DL. External shame and its association with depression and anxiety: the moderating role of self-compassion. Aust Psychol. (2021) 1–11. 10.1080/00050067.2021.1890984

[11] Shin SH Jiskrova GK Wills TA. Childhood maltreatment and alcohol use in young adulthood: the role of self-regulation processes. Addict Behav. (2019) 90:241–9. 10.1016/j.addbeh.2018.11.006 30471552

[12] Forgas JP Baumeister RF Tice DM. Psychology of Self-Regulations: Cognitive, Affective, and Motivational Processes: Chapter 1: The Psychology of Self-Regulation An Introductory Review. New York, NY: Psychology Press (2009).

[13] Lamia MC. Shame: A Concealed, Contagious, and Dangerous Emotion. Psychology Today (2011). Available online at: https://www.psychologytoday.com/us/blog/intense-emotions-and-strong-feelings/201104/shame-concealed-contagious-and-dangerous-emotion (accessed April 6, 2021).

[14] Velotti P Garofalo C Bottazzi F Caretti V. Faces of shame: implications for self-esteem, emotion regulation, aggression, and well-being. J Psychol. (2017) 151:171–84. 10.1080/00223980.2016.1248809 27858531

[15] Clark C Caldwell T Power C Stansfeld SA. Does the influence of childhood adversity on psychopathology persist across the lifecourse? A 45-year prospective epidemiologic study. Ann Epidemiol. (2010) 20:385–94. 10.1016/j.annepidem.2010.02.008 20382340

[16] Karatekin C. Adverse childhood experiences (ACEs), stress and mental health in college students. Stress Health. (2018) 34:36–45. 10.1002/smi.2761 28509376

[17] Schilling EA Aseltine RH Gore S. The impact of cumulative childhood adversity on young adult mental health: measures, models, and interpretations. Soc Sci Med. (2008) 66:1140–51. 10.1016/j.socscimed.2007.11.023 18177989 PMC2362506

[18] Mersky JP Topitzes J Reynolds AJ. Impacts of adverse childhood experiences on health, mental health, and substance use in early adulthood: a cohort study of an urban, minority sample in the U.S. Child Abuse Neglect. (2013) 37:917–25. 10.1016/j.chiabu.2013.07.011 23978575 PMC4090696

[19] Eshel N Daelmans B Mello MC Martines J. Responsive parenting: interventions and outcomes. Bull World Health Org. (2006) 84:992–9. 10.2471/BLT.06.030163 PMC2627571 17242836

[20] Whittle S Simmons JG Dennison M Vijayakumar N Schwartz O Yap MB. Positive parenting predicts the development of adolescent brain structure: a longitudinal study. Dev Cogn Neurosc. (2014) 8:7–17. 10.1016/j.dcn.2013.10.006 24269113 PMC6990097

[21] Clements-Nolle K Waddington R. Adverse childhood experiences and psychological distress in juvenile offenders: the protective influence of resilience and youth assets. J Adolesc Health. (2019) 64:49–55. 10.1016/j.jadohealth.2018.09.025 30579436

[22] Flaherty EG Thompson R Dubowitz H Harvey EM English DJ Proctor LJ. Adverse childhood experiences and child health in early adolescence. JAMA Pediatr. (2013) 167:622–9. 10.1001/jamapediatrics.2013.22 23645114 PMC3732117

[23] Shonkoff JP Cameron J Duncan G Fox NA Greenough W Gunnar MR. Children's Emotional Development Is Built into the Architecture of Their Brains: Working Paper No. 2. (2004). Available online at: https://developingchild.harvard.edu/wp-content/uploads/2004/04/Childrens-Emotional-Development-Is-Built-into-the-Architecture-of-Their-Brains.pdf (accessed April 6, 2021).

[24] Kessler RC Amminger GP Aguilar-Gaxiola S Alonso J Lee S Ustün TB. Age of onset of mental disorders: a review of recent literature. Curr Opin Psychiatry. (2007) 20:359–64. 10.1097/YCO.0b013e32816ebc8c 17551351 PMC1925038

[25] Niendam TA Laird AR Ray KL Dean YM Glahn DC Carter CS. Meta-analytic evidence for a superordinate cognitive control network subserving diverse executive functions. Cogn Affect Behav Neurosci. (2012) 12:241–68. 10.3758/s13415-011-0083-5 22282036 PMC3660731

[26] Center on the Developing Child at Harvard University. Building the Brain's “Air Traffic Control” System: How Early Experiences Shape the Development of Executive Function: Working Paper no. 11. (2011). Available online at: http://www.developingchild.harvard.edu (accessed April 6, 2021).

[27] Bastin C Harrison BJ Davey CG Moll J Whittle S. Feelings of shame, embarrassment and guilt and their neural correlates: a systematic review. Neurosci Biobehav Rev. (2016) 71:455–71. 10.1016/j.neubiorev.2016.09.019 27687818

[28] Deater-Deckard K. Family matters: intergenerational and interpersonal processes of executive function and attentive behavior. Curr Direct Psychol Sci. (2014) 23:230–6. 10.1177/0963721414531597 25197171 PMC4153697

[29] Barbey AK Colom R Solomon J Krueger F Forbes C Grafman J. An integrative architecture for general intelligence and executive function revealed by lesion mapping. Brain J Neurol. (2012) 135:1154–64. 10.1093/brain/aws021 22396393 PMC3326251

[30] Halfon N Shulman E Hochstein M. Brain Development in Early Childhood. (2001). Available online at: https://files.eric.ed.gov/fulltext/ED467320.pdf (accessed April 6, 2021).

[31] Luby JL Tillman R Barch DM. Association of timing of adverse childhood experiences and caregiver support with regionally specific brain development in adolescents. JAMA Network Open. (2019) 2:e1911426. 10.1001/jamanetworkopen.2019.11426 31532514 PMC6751767

[32] Coban AE. Interpersonal cognitive distortions and stress coping strategies of laate adolescents. Eurasian J Educ Res. (2013) 51:65–83.

[33] Mansfield CD Diamond LM. Does stress-related growth really matter for adolescents' day-to-day adaptive functioning? J Early Adolesc. (2017) 37:677–95. 10.1177/0272431615620665

[34] Fergus S Zimmerman MA. Adolescent resilience: a framework for understanding healthy development in the face of risk. Ann Rev Public Health. (2004) 26:399–419. 10.1146/annurev.publhealth.26.021304.144357 15760295

[35] Lackner CL Santesso DL Dywan J O'Leary DD Wade TJ Segalowitz SJ. Adverse childhood experiences are associated with self-regulation and the magnitude of the error-related negativity difference. Biol Psychol. (2018) 132:244–51. 10.1016/j.biopsycho.2018.01.006 29309827

[36] Crandall A Allsop Y Hanson CL. The longitudinal association between cognitive control capacities, suicidality, and depression during late adolescence and young adulthood. J Adolesc. (2018) 65:167–76. 10.1016/j.adolescence.2018.03.009 29602159

[37] Poole JC Dobson KS Pusch D. Childhood adversity and adult depression: the protective role of psychological resilience. Child Abuse Neglect. (2017) 64:89–100. 10.1016/j.chiabu.2016.12.012 28056359

[38] Kim J Talbot NL Cicchetti D. Childhood abuse and current interpersonal conflict: the role of shame. Child Abuse Neglect. (2009) 33:362–71. 10.1016/j.chiabu.2008.10.003 19457556 PMC4115782

[39] Schimmenti A. Unveiling the hidden self: developmental trauma and pathological shame. Psychodyn Pract. (2012) 18:195–211. 10.1080/14753634.2012.664873

[40] Stuewig J McCloskey LA. The relation of child maltreatment to shame and guilt among adolescents: psychological routes to depression and delinquency. Child Maltreatment. (2005) 10:324–36. 10.1177/1077559505279308 16204735

[41] Castilho P Carvalho SA Marques S Pinto-Gouveia J. Self-compassion and emotional intelligence in adolescence: a multigroup mediational study of the impact of shame memories on depressive symptoms. J Child Fam Stud. (2017) 26:759–68. 10.1007/s10826-016-0613-4

[42] Matos M Pinto-Gouveia J Duarte C. Internalizing early memories of shame and lack of safeness and warmth: the mediating role of shame on depression. Behav Cogn Psychother. (2013) 41:479–93. 10.1017/S1352465812001099 23522753

[43] Heatherton TF Wagner DD. Cognitive neuroscience of self-regulation failure. Trends Cogn Sci. (2011) 15:132–9. 10.1016/j.tics.2010.12.005 21273114 PMC3062191

[44] Persons E Kershaw T Sikkema KJ Hansen NB. The impact of shame on health-related quality of life among HIV-positive adults with a history of childhood sexual abuse. AIDS Patient Care STDs. (2010) 24:571–80. 10.1089/apc.2009.0209 20718687 PMC2957629

[45] Novak SP Clayton RR. The influence of school environment and self-regulation on transitions between stages of cigarette smoking: a multilevel analysis. Health Psychol. (2001) 20:196–207. 10.1037/0278-6133.20.3.196 11403217

[46] Crandall A Magnusson BM Novilla MLB Novilla LKB Dyer WJ. Family financial stress and adolescent sexual risk-taking: the role of self-regulation. J Youth Adolesc. (2017) 46:45–62. 10.1007/s10964-016-0543-x 27460827

[47] Harper JM Hoopes MH. Uncovering Shame: An Approach Integrating Individuals and Their Family Systems. New York, NY: WW Norton and Co. (1990).

[48] Weissman MM Orvaschel H Padian N. Children's symptom and social functioning self-report scales. Comparison of mothers' and children's reports. J Nerv Ment Dis. (1980) 168:736–40. 10.1097/00005053-198012000-00005 7452212

[49] Spence SH. A measure of anxiety symptoms among children. Behav Res Ther. (1998) 36:545–66. 10.1016/S0005-7967(98)00034-5 9648330

[50] Moberg DP Hahn L. The adolescent drug involvement scale. J Adolesc Chem Depend. (1991) 2:75–88. 10.1080/10678289109512338

[51] Bruner C. ACE, place, race, and poverty: building hope for children. Academic Pediatr. (2017) 17:S123–9. 10.1016/j.acap.2017.05.009 28865644

[52] Sacks V Murphey D. The Prevalence of Adverse Childhood Experiences, Nationally, by State, and by Race or Ethnicity. Bethesda, MD: Child Trends (2018).

[53] Preacher KJ Hayes AF. Asymptotic and resampling strategies for assessing and comparing indirect effects in multiple mediator models. Behav Res Methods. (2008) 40:879–91. 10.3758/BRM.40.3.879 18697684

[54] Hu L Bentler PM. Cutoff criteria for fit indexes in covariance structure analysis: conventional criteria versus new alternatives. Struct Equ Model Multidiscip J. (1999) 6:1–55. 10.1080/10705519909540118

[55] Spenrath MA Clarke ME Kutcher S. The science of brain and biological development: implications for mental health research, practice and policy. J Can Acad Child Adolesc Psychiatry. (2011) 20:298–304. 22114611 PMC3222573

[56] Strohmeier CW Rosenfield B DiTomasso RA Ramsay JR. Assessment of the relationship between self-reported cognitive distortions and adult ADHD, anxiety, depression, and hopelessness. Psychiatry Res. (2016) 238:153–8. 10.1016/j.psychres.2016.02.034 27086226

[57] Reimann Z Miller JR Dahle KM Hooper AP Young AM Goates MC Crandall A. Executive functions and health behaviors associated with the leading causes of death in the United States: a systematic review. J Health Psychol. (2018) 25:186–96. 10.1177/1359105318800829 30230381

[58] Sedighimornani N Rimes KA Verplanken B. Exploring the relationships between mindfulness, self-compassion, and shame. SAGE Open. (2019) 9:2158244019866294. 10.1177/2158244019866294

[59] Pandey A Hale D Das S Goddings A-L Blakemore S-J Viner RM. Effectiveness of universal self-regulation-based interventions in children and adolescents: a systematic review and meta-analysis. JAMA Pediatr. (2018) 172:566–75. 10.1001/jamapediatrics.2018.0232 29710097 PMC6059379

[60] Fosco GM Frank JL Stormshak EA Dishion TJ. Opening the “Black Box”: family check-up intervention effects on self-regulation that prevents growth in problem behavior and substance use. J Sch Psychol. (2013) 51:455–68. 10.1016/j.jsp.2013.02.001 23870441 PMC3717583

[61] Stormshak EA Fosco GM Dishion TJ. Implementing interventions with families in schools to increase youth school engagement: the family check-up model. Sch Mental Health. (2010) 2:82–92. 10.1007/s12310-009-9025-6 20495673 PMC2873213

[62] Lemberger ME Selig JP Bowers H Rogers JE. Effects of the student success skills program on executive functioning skills, feelings of connectedness, and academic achievement in a predominantly hispanic, low-income middle school district. J Counsel Dev. (2015) 93:25–37. 10.1002/j.1556-6676.2015.00178.x

